# Molecular Mechanism of m6A Methylation Modification Genes *METTL3* and *FTO* in Regulating Heat Stress in Sheep

**DOI:** 10.3390/ijms241511926

**Published:** 2023-07-25

**Authors:** Bowen Chen, Chao Yuan, Tingting Guo, Jianbin Liu, Bohui Yang, Zengkui Lu

**Affiliations:** 1 Key Laboratory of Animal Genetics and Breeding on the Tibetan Plateau, Ministry of Agriculture and Rural Affairs, Lanzhou Institute of Husbandry and Pharmaceutical Sciences, Chinese Academy of Agricultural Sciences, Lanzhou 730050, China; chenbw202204@163.com (B.C.); yuanchao@caas.cn (C.Y.); guotingting@caas.cn (T.G.); liujianbin@caas.cn (J.L.); 2 Sheep Breeding Engineering Technology Research Center of Chinese Academy of Agricultural Sciences, Lanzhou 730050, China

**Keywords:** heat stress, m6A methylation, *METTL3*, *FTO*, sheep

## Abstract

Heat stress is an important environmental factor affecting livestock production worldwide. Primary hepatocytes and preadipocytes derived from Hu sheep were used to establish a heat stress model. Quantitative reverse transcriptase-PCR (qRT-PCR) analysis showed that heat induction significantly increased the expression levels of heat stress protein (HSP) genes and the N^6^-methyladenosine (m6A) methylation modification genes: methyltransferase-like protein 3 (*METTL3*), methyltransferase-like protein 14 (*METTL14*), and fat mass and obesity associated protein (*FTO*). Heat stress simultaneously promoted cell apoptosis. Transcriptome sequencing identified 3980 upregulated genes and 2420 downregulated genes related to heat stress. A pathway enrichment analysis of these genes revealed significant enrichment in fatty acid biosynthesis, degradation, and the PI3K-Akt and peroxisome proliferator-activated receptor (PPAR) signaling pathways. Overexpression of *METTL3* in primary hepatocytes led to significant downregulation of *HSP60*, *HSP70*, and *HSP110*, and significantly increased mRNA m6A methylation; *FTO* interference generated the opposite results. Primary adipocytes showed similar results. Transcriptome analysis of cells under *METTL3* (or *FTO*) inference and overexpression revealed differentially expressed genes enriched in the mitogen-activated protein kinase (MAPK) signaling pathways, as well as the PI3K-Akt and Ras signaling pathways. We speculate that *METTL3* may increase the level of m6A methylation to inhibit fat deposition and/or inhibit the expression of HSP genes to enhance the body’s resistance to heat stress, while the *FTO* gene generated the opposite molecular mechanism. This study provides a scientific basis and theoretical support for sheep feeding and management practices during heat stress.

## 1. Introduction

Sheep are innately fearful of damp heat, which makes them vulnerable to heat stress. According to the report by the Intergovernmental Panel on Climate Change [1], global warming has increased the frequency and intensity of extreme high-temperature events, exacerbating heat stress in sheep. Furthermore, the practice of intensive high-density feeding of sheep in China has made them more susceptible to heat stress (HS). Similar to pigs and poultry, sheep have a specific range of temperature adaptation, with the optimal temperature ranging from −3 °C to 23 °C. Although sheep can tolerate a wide range of temperatures and have high heat resistance, when the ambient temperature exceeds the optimal range, their body temperature and metabolic heat production increase, leading to heat stress [2]. This can cause an increase in respiratory rate and hyperventilation, resulting in respiratory alkalosis. The body compensates for this by excreting bicarbonate in the urine, leading to a decrease in blood pH and metabolic acidosis, which results in the accumulation of pyruvate in the muscle [3]. Heat stress can also cause increased mortality and reductions in growth, fertility, milk production, and lambing rates in these animals [4]. Heat stress not only seriously affects the productivity, product quality, and health index of sheep, but it also increases veterinary costs, husbandry management expenses, and animal welfare problems [3,5]. Therefore, research on the molecular mechanism of the response to heat stress in sheep is urgently important.

N^6^-methyladenosine (m6A) methylation is the most abundant epigenetic RNA modification in eukaryotic cells [6,7]. This dynamic and reversible process, which involves the deposition, removal, and recognition of m6A, is induced by methyltransferases, namely methyltransferase-like protein 3 (METTL3), methyltransferase-like protein 14 (METTL14), and wilms tumor 1 associated protein (WTAP); demethylases, namely fat mass and obesity associated protein (FTO) and Alk B homologue 5 (ALKBK5); and m6A-binding proteins, namely YTH domain family proteins 1 (YTHDF1), YTHDF2, YTH domain containing proteins 1 (YTHDC1), and YTHDC2 [8]. These enzymes are involved in almost all RNA metabolic processes, including mRNA cleavage, stability, and translation, and play a decisive role in the fate of RNA [8,9,10]. In mouse embryonic fibroblasts, adenosine in the 5′-untranslated region (UTR) is preferentially methylated in response to heat stress [11]. The peak of m6A modification in the 5′-UTR is due to the nuclear localization of m6A reader protein YTHDF2 following stress induction. Under heat stress, nuclear YTHDF2 maintains the methylation of the 5′-UTR of stress-induced transcripts by limiting the demethylation activity of FTO [12]. Additionally, heat stress-induced translation of heat shock protein (HSP)70 is mediated by the 5′-UTR, which contain m6A modification sites. Under heat stress, the m6A modification peak of the *HSP70* 5′-UTR was significantly increased in HeLa cells, allowing it to be translated in a cap-independent manner [13]. Those finding demonstrated that m6A methylation modification is involved in regulating the heat stress response of animals.

Our previous studies have shown that the expression levels of *METTL3*, *METTL14*, *WTAP*, *FTO*, *ALKBH5*, *YTHDF1*, *YTHDF2*, *YTHDF3*, *YTHDC1*, and *YTHDC2* in sheep liver tissue were significantly upregulated under heat stress [14], indicating that heat stress affects m6A methylation in sheep liver tissue. Additionally, m6A methylation modification genes were significantly enriched in pathways related to fat metabolism, such as Wnt, transforming growth factor-β, and AMP-activated protein kinase [15]. These results revealed that m6A methylation modification may play an important role in liver fat metabolism. Adipocytes are the most important cells for fat metabolism. Therefore, this study aimed to establish in vitro heat stress models of primary liver cells and preadipocytes derived from Hu sheep to elucidate the molecular mechanism of m6A methylation modification genes regulating heat stress in sheep. We examined the effects of RNA interference (RNAi) and overexpression of the m6A writer *METTL3* and the demethylase *FTO* using quantitative reverse transcriptase-PCR (qRT-PCR), indirect immunofluorescence, and transcriptome sequencing. Our findings provide a scientific basis and theoretical support in sheep feeding and management practices during heat stress periods.

## 2. Results

### 2.1. Cell Culture and Identification

The culture and identification of sheep primary hepatocytes were performed as previously described [16]. Briefly, primary hepatocytes presented with irregular polygon morphology after adherent growth. After periodic acid–Schiff staining for glycogen and assessment of alpha-fetoprotein (AFP) expression, the isolated cells were identified as hepatocytes using the detection of hepatocyte-specific markers (cytokeratin (CK)-18 and albumin). Cell purity was at least 95% based on CK-18, AFP, and albumin staining [16].

Soon after inoculation, primary adipocytes were suspended, small, and round-shaped. After 24 h of incubation, the cells began to adhere and grow. The adherent cells were mostly irregularly or diamond-shaped. After 48 h of culture, all of the cells were adherent, and most were spindle-shaped. After 4 days of culture, the cells entered the rapid growth phase, and the cell density increased (Figure S1A). Oil red O staining showed that small lipid droplets had appeared in some adipocytes that had grown to monolayer confluence (Figure S1B), indicating that the isolated preadipocytes had the ability to proliferate and differentiate. Cell Counting Kit-8 (CCK-8) analysis showed that the optical density (OD) value increased with the prolongation of culture time, indicating that adipocytes continued to proliferate during culture, with the fastest proliferation time being 5–7 days. Primary adipocytes cultured in two different media both showed normal proliferation ability (Figure S1C). However, the OD value of primary adipocytes cultured in Dulbecco’s modified Eagle’s medium (DMEM)/F12 + 10% fetal bovine serum (FBS) was significantly higher than that in DMEM + 10% FBS, indicating that DMEM/F12 + 10% FBS was more suitable for the rapid proliferation and culture of primary adipocytes.

### 2.2. Establishment of an In Vitro Heat Stress Model in Primary Hepatocytes and Adipocytes

**Figure 1:**
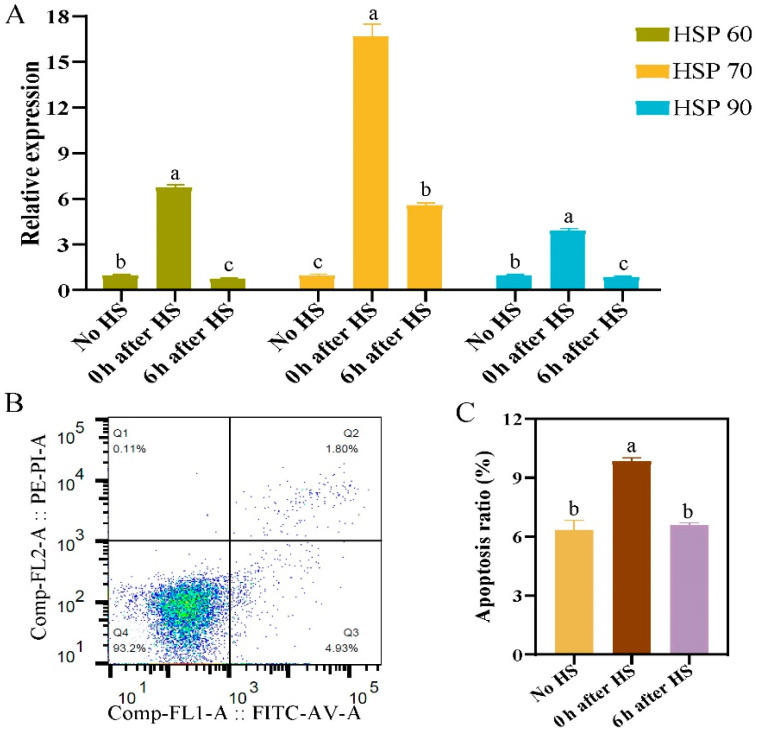
Detection of heat stress proteins genes and apoptosis rate of primary hepatocytes. (**A**) Quantitative reverse transcription-PCR analysis of relative expression of HSP genes *HSP60*, *HSP70*, and *HSP90*. (**B**) Schematic diagram of flow cytometry of the apoptosis ratio in primary hepatocytes with no HS. (**C**) Bar chart of apoptosis ratio of primary hepatocytes with and without HS. Groups marked with the same letter were considered to have no significant difference, and those without the same letter were significantly different (*p* < 0.05).

According to a report by Yu et al. [17,18], 42 °C is the ideal temperature to induce heat stress in primary hepatocytes and preadipocytes. Figure 1A shows that the expression of heat stress genes *HSP60*, *HSP70*, and *HSP90* in hepatocytes was significantly increased immediately following a 1 h heat stress treatment (0 h time point) compared with no heat stress (*p* < 0.05), and was significantly decreased following a 6 h recovery (6 h time point) (*p* < 0.05). The apoptosis of primary hepatocytes after heat stress was detected by flow cytometry, which showed that the ratio of apoptosis rate reached 9.85% at 0 h and decreased significantly at 6 h post heat stress (Figure 1B,C). Therefore, a 0 h recovery was selected as the best time point in which to examine the effects of heat stress on primary hepatocytes.

Due to the different degree of heat sensitivity of primary adipocytes, the heat stress time was selected according to the report [19,20]. The expression levels of *HSP60*, *HSP70*, and *HSP90* in primary adipocytes after heat stress for 1, 2, or 3 days were also examined using qRT-PCR. The results showed that *HSP60* and *HSP90* expression increased with the duration of heat stress, whereas *HSP70* expression was the highest at day 2 of heat stress, and then decreased thereafter (*p* < 0.05) (Figure S2A). The apoptosis ratio in primary adipocytes did not change the prolongation of heat stress (Figure S2B,C). Therefore, day 2 was selected as the best duration of heat stress for primary adipocytes.

### 2.3. Effect of Heat Stress on Expression of Methylation-Related Factors

**Figure 2:**
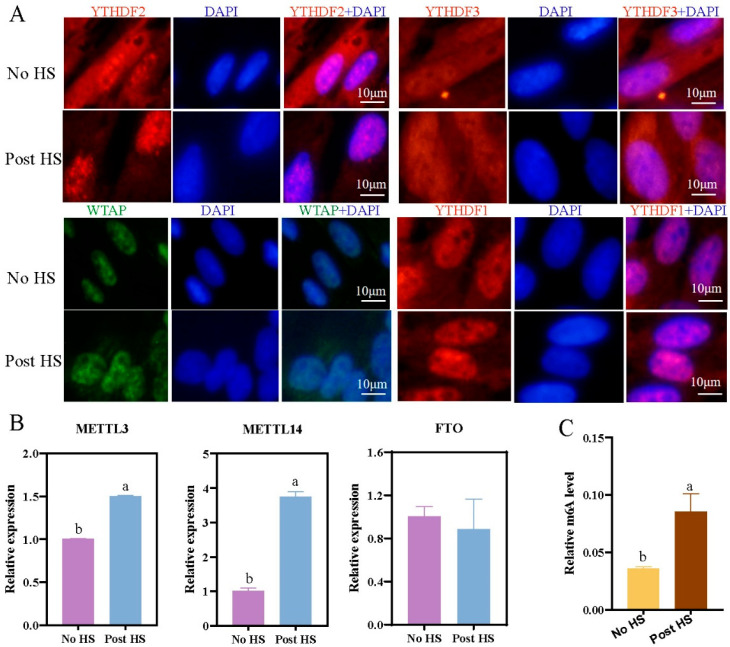
Changes in expression of methylation-related genes and m6A methylation levels with and without HS in primary hepatocytes. (**A**) Immunofluorescence assays of YTHDF1, YTHDF2, YTHDF3, and WTAP with no HS and at 0 h post HS. DAPI: 4′,6-diamidino-2-phenylindole stain. YTHDF2, YTHDF3, and YTHDF1 are indicated with red fluorescence, WTAP is green fluorescence and DAPI with blue. (**B**) qRT-PCR analysis of relative expression of *METTL3*, *METTL14*, and *FTO* expression under no HS and at 0 h post HS. (**C**) Quantification mRNA m6A-methylated under no HS and at 0 h post HS. DAPI: 4′,6-diamidino-2-phenylindole stain. Groups marked with the same letter were considered to have no significant difference, and those without the same letter were significantly different (*p* < 0.05).

Immunofluorescence assays showed that YTHDF2 was transferred from the cytoplasm to the nucleus after heat stress in hepatocytes (Figure 2A). YTHDF3 and YTHDC2 were detected in both cytoplasm and nuclei. YTHDF1, WTAP, METTL3, METTL14, ALKBH5, FTO, and YTHDC1 were all found in the nucleus, and heat stress did not affect their localization (Figure S3). Additionally, qRT-PCR analysis showed that heat stress upregulated the expression of *METTL3* and *METTL14* (*p* < 0.05), but had no effect on *FTO* expression (Figure 2B). However, the m6A methylation level in hepatocytes induced with heat stress was significantly increased compared with that in uninduced hepatocytes (*p* < 0.05) (Figure 2C). Consistent with primary hepatocytes, primary preadipocytes subjected to heat stress showed upregulated expression of the methylation-related genes *METTL3*, *METTL14*, *FTO*, and *YTHDF3* and significantly increased m6A methylation (Figure S4A,B).

### 2.4. Identification of Heat-Stress-Related Differentially Expressed Genes (DEGs) and Functional Enrichment Analysis of Primary Hepatocytes

**Figure 3:**
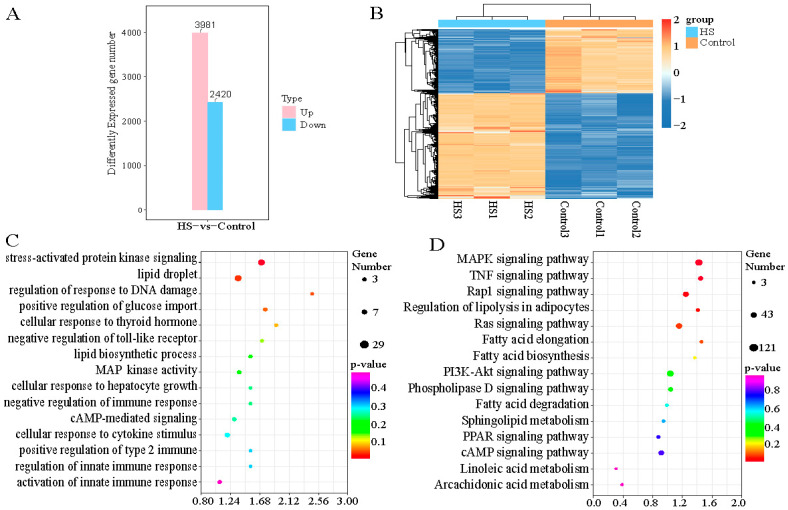
Transcriptomic comparisons and functional enrichment analysis of the HS and no HS (Control) groups of primary hepatocytes. (**A**) Statistical distribution of DEGs showing upregulation and downregulation. (**B**) Heat map of cluster analysis of DEGs. (**C**) GO annotation (**D**) and KEGG enrichment analysis of DEGs, with GO terms and KEGG pathways indicated on the ordinate axes, respectively.

To further understand the metabolic pathways involved in heat stress regulation, we performed transcriptome RNA sequencing (RNA-seq) analysis of primary hepatocytes with no heat stress (37 °C control) and at 0 h post heat stress (42 °C). After high-throughput sequencing and filtering the raw reads for quality control, we obtained 142.16 and 146.09 million high-quality, clean reads for the control and heat shock groups, respectively (Supplementary Table S1). For each sample, >95% of the reads could be mapped to the reference sheep genome (Oar. v4.0, https://www.ncbi.nlm.nih.gov/assembly/GCF_000298735.2/, accessed on 10 May 2023). We obtained 6401 DEGs between the heat shock and control groups (Supplementary Table S2), comprising 3981 upregulated and 2420 downregulated genes (Figure 3A). Two-way cluster analysis was undertaken to examine the expression patterns of the heat stress-related DEGs in hepatocytes. This analysis revealed that the DEGs formed a single cluster, while the differentially upregulated genes and downregulated genes in each group comprised separate clusters (Figure 3B). To understand the functions of these DEGs, we conducted Gene Ontology (GO) functional enrichment analysis, which yielded 5018 enriched GO terms, including 3258 biological process terms, 1031 molecular function terms, and 729 cellular component terms. These GO terms were involved in stimulus response (stress-activated protein kinase signaling cascade, DNA damage stimulus, thyroid hormone stimulus, and cAMP-mediated signaling), immune response (innate immune response), and fat metabolism (lipid droplet, lipid biosynthetic process, and mitogen-activated protein kinase (MAPK) activity) (Figure 3C). Kyoto Encyclopedia of Genes and Genomes (KEGG) pathway analysis revealed that these differential genes were significantly enriched in 94 pathways (*p* < 0.05), and further analysis revealed that many of these pathways were related to fat metabolism, including fatty acid biosynthesis, elongation, degradation, arachidonic acid metabolism, PI3K-Akt signaling, and peroxisome proliferator activated receptor (PPAR) signaling pathways. Signaling pathways that regulate the stress response were also enriched, namely, the Ras, Rap1, and tumor necrosis factor (TNF) signaling pathways (Figure 3D).

### 2.5. Molecular Mechanism of METTL3 in the Regulation of Heat Stress

We used qRT-PCR to confirm that *METTL3* expression was significantly increased following infection of primary hepatocytes with a lentiviral *METTL3* overexpression construct (*p* < 0.05). Conversely, infection with a lentiviral *METTL3* RNAi construct led to significantly decreased *METTL3* expression (*p* < 0.05). The results of *METTL3* lentivirus infections of primary adipocytes were similar (Figure S5).

**Figure 4:**
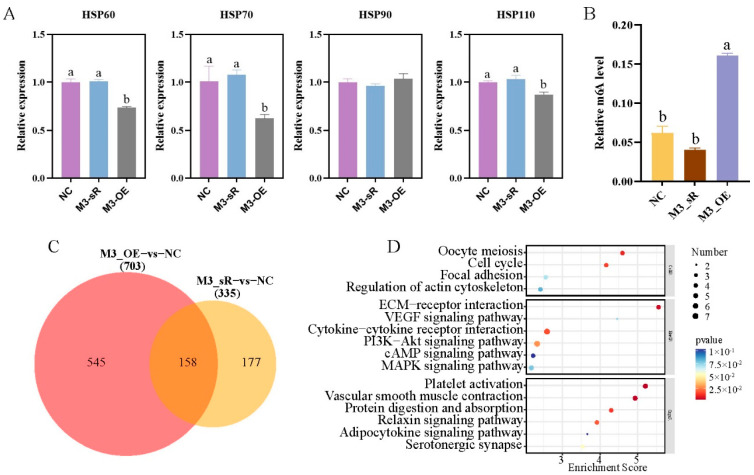
Effects of *METTL3* interference and overexpression on heat stress in primary hepatocytes. (**A**) qRT-PCR analysis of *HSP60*, *HSP70*, *HSP90*, and *HSP110* expression in NC group and in cells infected with the lentiviral *METTL3* interference (M3_sR) and overexpression (M3_OE) constructs. (**B**) Quantification of mRNA m6A methylation in the NC, M3_sR, and M3_OE groups. (**C**) Venn analysis of DEGs in the *METTL3* interference and overexpression groups. (**D**) KEGG enrichment analysis of DEGs shared between the two groups. Groups marked with the same letter were considered to have no significant difference, and those without the same letter were significantly different (*p* < 0.05).

Compared with negative control (NC), expression levels of *HSP60*, *HSP70*, *HSP90*, and *HSP110* did not change after *METTL3* interference (Figure 4A). By contrast, expression levels of *HSP60*, *HSP70*, and *HSP110* significantly decreased under *METTL3* overexpression compared with those in negative control (Figure 4A), indicating that the overexpression of *METTL3* significantly inhibited the expression of heat stress genes. The m6A methylation level significantly increased after *METTL3* overexpression (*p* < 0.05), and decreased after *METTL3* interference, although not significantly (Figure 4B). Similarly, in primary preadipocytes, the expression of *HSP70* and *HSP90* significantly decreased, while the m6A methylation level significantly increased, following *METTL3* overexpression (Figure S6A,B).

To further understand the changes in signaling pathways involved in *METTL3* interference and overexpression, we compared RNA-seq results in primary hepatocytes under *METTL3* interference versus overexpression. Compared with negative control, 142 differentially upregulated genes and 193 differentially downregulated genes were screened in the *METTL3* interference group, while 338 differentially upregulated genes and 365 differentially downregulated genes were screened in the *METTL3* overexpression group (*p* < 0.05) (Supplementary Table S3). Venn analysis revealed that the two groups shared 158 DEGs (Figure 4C). KEGG enrichment analysis of these common DEGs showed that most were enriched in the PI3K-Akt, cAMP, and MAPK signaling pathways (Figure 4D).

### 2.6. Molecular Mechanism of FTO in the Regulation of Heat Stress

The results of qRT-PCR showed that *FTO* expression was significantly increased following infection of primary hepatocytes with a lentiviral *FTO* overexpression construct. Conversely, infection with a lentiviral *FTO* RNAi construct led to significantly decreased *FTO* expression (Figure S5). The results of *FTO* lentivirus infections of primary adipocytes were similar.

**Figure 5:**
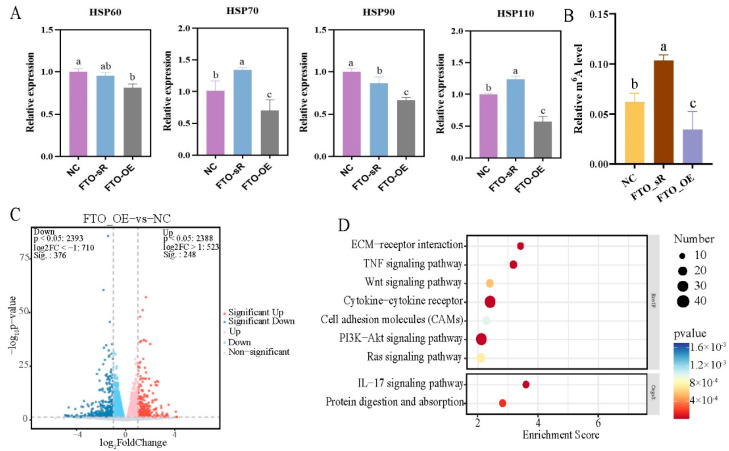
Effects of *FTO* interference and overexpression on heat stress in primary hepatocytes (**A**) qRT-PCR analysis of *HSP60*, *HSP70*, *HSP90*, and *HSP110* expression in NC group, and in cells infected with the lentiviral *FTO* interference (FTO_sR) and overexpression (FTO_OE) constructs. (**B**) Quantification mRNA m6A methylation in the NC, FTO_sR, and FTO_OE groups. (**C**) Heat map of cluster analysis of DEGs. (**D**) KEGG enrichment analysis of DEGs shared between the two groups. Groups marked with the same letter were considered to have no significant difference, and those without the same letter were significantly different (*p* < 0.05).

qRT-PCR analysis showed that expression levels of *HSP70* and *HSP110* were significantly upregulated following *FTO* interference (*p* < 0.05), while expression levels of *HSP60*, *HSP70*, *HSP90*, and *HSP110* were significantly decreased following *FTO* overexpression (Figure 5A) in primary hepatocytes. The m6A methylation level increased significantly after *FTO* interference, and decreased after *FTO* overexpression (Figure 5B). Similar results were observed in primary preadipocytes: the m6A methylation level increased significantly after *FTO* interference and decreased after *FTO* overexpression (Figure S6C,D).

To further understands the changes in signaling pathways involved in *FTO* interference and overexpression, we compared RNA-seq results in primary hepatocytes under *FTO* interference and overexpression. Compared with the NC group, 255 differentially upregulated genes and 185 differentially downregulated genes were screened in the *FTO* interference group, while 248 differentially upregulated genes and 376 differentially downregulated genes were screened in the *FTO* overexpression group (*p* < 0.05) (Figure 5C, Supplementary Table S4). Venn analysis revealed that the two groups shared 126 DEGs. KEGG enrichment analysis of these common DEGs showed that most were enriched in the cytokine–cytokine receptor, PI3K-Akt, and Ras signaling pathways (Figure 5D).

## 3. Discussion

Temperature is the most important ecological factor affecting sheep production. As the frequency of extreme high-temperature weather events gradually increases because of global warming, the impacts of heat stress on sheep are becoming more and more prominent. Heat stress can cause rates of feed intake and feed utilization in livestock animals to decline, which eventually leads to decreased animal production performance and immune function and increased mortality, resulting in huge economic losses and problems maintaining animal welfare [21,22,23,24]. Consequently, an increasing amount research is focused on the alleviation of the heat stress response in animals to reduce its impact on livestock production, which is of great importance to the healthy development of the animal husbandry industry.

Previous studies have found that the expression of *METTL3*, *METTL14*, *WTAP*, *FTO*, and other methylation modification genes were significantly upregulated in sheep liver tissue following heat stress, indicating that m6A methylation modification genes may play an important role in the regulation of heat stress in liver cells [14,15]. In this study, primary hepatocytes were isolated and cultured and used to establish a heat stress model (Figure 1). We found that primary hepatocytes had similar results after heat stress. Additionally, the localization of YTHDF2 was transferred from the cytoplasm to the nucleus, indicating that heat stress promoted the transcription of *YTHDF2*, which is consistent with the results of previous studies [12,17,25]. Because adipocytes are the key fat-metabolizing cells, we verified our results in sheep adipocytes. Heat-stress-induced changes in the expression of m6A methylation modification genes in adipocytes were consistent with the results in primary hepatocytes, indicating that heat stress can induce responses in metabolic organs in animals [26,27]. To verify the regulatory mechanism of m6A methylation modification genes on the heat stress response in liver cells and adipocytes, we examined the effects of interference and overexpression of *METTL3* and *FTO* on the expression of HSP genes in these cells. Although the HSP genes all showed significantly decreased expression under overexpression of either *METTL3* or *FTO*, the expression patterns differed under the interference of *METTL3* or *FTO* (Figure 4A and Figure 5A). Similarly, Yu et al. found that *METTL3* knockdown using small interfering RNA significantly increased mRNA expression of *HSP70*, *HSP60*, and *HSP27* in HepG2 cells, whereas the expression of *HSP60* was significantly reduced. In their study, no differences were observed for *HSP90AA1* and *HSF1* mRNA [17]. We suspect that these differences may be associated with the positions and overall abundance of m6A sites on HSP gene transcripts; however, further research is needed.

Heat stress is one of the major challenges to livestock productivity because it can lead to changes in energy metabolism and body-wide immunity. High-throughput sequencing technology has been shown to be useful in the detection of changes in molecular regulatory and metabolic pathways in cows, pigs, chickens, and other animals [28,29,30]. Therefore, we used transcriptomics to screen for gene expression changes in sheep hepatocytes following heat stress. The identified DEGs included stress-response-related genes, namely mitogen-activated protein kinase 3 (*MAP2K3*), *MAP2K6*, *MAP2K7*, and *MAP3K1*; DNA damage and repair genes, namely poly polymerase family member 3 (*PARP3*), PARP1 binding protein (*PARPBP*), dual specificity phosphatase 1 (*USP1*), *USP28*, and *USP45*; and cell apoptosis genes, namely actin gamma 1 (*ACTG1*), AKT serine/threonine kinase 1 (*AKT1*), BCL2 like 1 (*BCL2L1*), and beclin 1 (*BECN1*). KEGG enrichment analysis revealed that some genes were significantly enriched in stress-related signaling pathways (e.g., Rap1, MAPK, and PI3K-Akt), as well as pathways related to fat metabolism (e.g., MAPK, PI3K-Akt, and cAMP) [31,32,33,34,35]. Also included were pathways related to the regulation of immune responses [36]. Rap1, as a molecular switch for stress generation, is regulated by a large number of external stimuli. The MAPK signaling pathway can be activated by a variety of external stimuli and is stimulated by Rap1 to play a role in a variety of cell types [32]. Similarly, the PI3K-Akt pathway is also activated by many types of extracellular stimuli and has been shown to have a cascade reaction with the MAPK signaling pathway [31]. Taken together, these stress-related signaling pathways may form a complex cascade response to reduce the damage caused by heat stress in sheep. The study also found that the expression of lipid accumulation related genes was up-regulated after heat stress in primary hepatocytes, such as acyl-CoA dehydrogenase family member 11 (*ACAD11*), ATP binding cassette subfamily G member 5 (*ABCG5*), fatty acid binding protein 4 (*FABP4*), etc. [37], indicating that heat stress can promote adipogenesis [38]. Zhong et al. found that *METTL3* participates in animal fat metabolism by regulating certain fat metabolism pathways and adipose-tissue-specific gene expression [39,40]. The results of this study showed that the methylation level of m6A increased after overexpression of *METTL3* in primary hepatocytes and led to significantly decreased expression of HSP genes, but some genes related to fatty acid metabolism [41], such as a gene negatively regulating fatty acid synthesis—(BRCA1 DNA repair associated (*BRCA1*)), was down-regulated, and a gene involved in fatty acid metabolic process—(gamma-glutamyltransferase 5 (*GGT5*)) was increased significantly. Those results indicated that *METTL3* may increase the level of m6A methylation to inhibit fat deposition (or promote fat metabolism) and/or inhibit the expression of HSP genes to enhance the body’s resistance to heat stress. Interestingly, the methylation level of m6A decreased after the overexpression of *FTO* in primary hepatocytes, and the expression of lipid-accumulation-related genes was up-regulated [42], such as adiponectin, C1Q and collagen domain containing genes (*ADIPOQ*) and *FABP4*, indicating that the overexpression of *FTO* increases fat deposition by reducing the level of m6A.

Heat stress is one of the major challenges to livestock productivity [43]. In order to cope with and solve the impact of heat stress on animal husbandry, in recent years, domestic and foreign scholars have mainly solved the problem of heat stress in animals from the aspects of physical methods and nutritional regulation. Physical cooling mainly includes rational construction of enclosures and the improvement of ventilation and cooling equipment in enclosures, but these measures cannot fundamentally solve the problem; meanwhile, nutritional regulation is more economical, convenient, and easy to promote and implement efficiently and has become an effective way to alleviate heat stress in animals [44,45]. Nutrition regulation mainly includes adjusting the nutrient level of the diet and adding anti-heat stress substances to adjust the heat shock level and immune function of animals, so as to alleviate the heat stress of animals. For example, adding methyl donor-betaine to feed can effectively alleviate the heat stress of dairy cows and zebrafish [46,47]. The results of this study also found that the expression of heat shock proteins can be significantly reduced after the overexpression of *METTL3* and *FTO*. This result is consistent with previous studies [17]. Because m6A plays vital roles in adipogenesis, and studies have shown that adding a certain amount of methyl donor-betaine can increase methylation levels and inhibited lipogenesis [48,49], therefore, we speculate that *METTL3* or *FTO* genes may regulate sheep heat stress through fat metabolism. However, the specific molecular mechanism needs to be further explored. These results further a provide scientific basis and theoretical support for adding betaine to sheep feed to relieve heat stress.

## 4. Materials and Methods

### 4.1. Cell Isolation and Culture Procedures

Three one-day-old newborn sheep (Hu sheep, 1.5–3 kg, ♂) from Lanzhou Wanshan Plantation and Breeding Professional Cooperative were used in this study. Those sheep were anesthetized with isoflurane inhalation (Sigma-Aldrich, St. Louis, MO, USA) and then fixed on a trough-shaped stool, and the carotid artery was cut off for bloodletting and slaughter. After the bloodletting was completed, use a sterile sharp knife to pick up the sheepskin along the midline of the abdomen, the livers and perirenal, subcutaneous adipose tissue were obtained and immersed in normal saline containing 2% penicillin–streptomycin (PS, Gibco, Carlsbad, CA, USA) and then brought to the laboratory.

Specific separation method of sheep primary hepatocytes was referenced to [16]. Sterile scissors were used to cut 1 × 1 mm^3^ liver tissues after the liver tissues were washed with 1 × phosphate buffered saline (PBS, Solarbio, Beijing, China). Then, 5 mL 0.25% trypsin (Gibco) and 0.1 mg/mL type IV collagenase (Sigma-Aldrich) in a ratio of 1:1 was added for digestion and incubated at 37 °C in a water bath for 15 min with continuous shaking until the digestion was complete. After the digestion, a complete medium was added to stop the digestion, and the cells were filtered using a 100 μm sieve. The cell suspension was centrifuged at 800 rpm for 5 min, and the supernatant was discarded, resuspended in 3 mL ACK lysis buffer (Solarbio), and incubated at room temperature for 10 min. The cells were again centrifuged at 800 rpm for 5 min and the supernatant was discarded, followed by resuspension in a complete medium (William’s Medium E (Gibco) containing 15% FBS (Gibco)) and culturing in 6-well plates. Finally, the non-adherent hepatocytes and dead cells were discarded the next day, and 2 mL of new complete medium was added to the 6-well plate, coated with rat tail collagen, and incubated at 37 °C in a 5% CO_2_ incubator.

The separation steps to obtain primary preadipocytes were as follows. Adipose tissue was cut into 1 mm^3^ tissue blocks with sterile scissors after washing three times with PBS and then digested with 1 mg/mL type I collagenase (Sigma-Aldrich) at 37 °C for 60–90 min. The supernatant was discarded after filtration with a 100 μm cell sieve and centrifuged at 1500 rpm for 10 min. The cells were resuspended, passed through a 70 μm cell sieve, centrifuged at 1500 rpm/min for 5 min, resuspended, and counted. 6-well culture plates were inoculated with the cells at a density of 5 × 10^4^/cm^2^ and cultured in a 5% CO_2_ incubator at 37 °C. The cultures were tested and confirmed to be negative for mycoplasma contamination before use.

### 4.2. Cell Proliferation and Apoptosis Detection

CCK-8 (ZETA LIFE, Menlo Park, CA, USA) was used to monitor cell proliferation per the manufacturer’s instructions. Approximately 2 × 10^3^ cells were seeded into a 96-well plate, 10 μL CCK-8 solution was added to each well, and the cells were incubated at 37 °C for 2 h. The OD was then measured using a Multiskan FC-type microplate reader at 450 nm (n = 3, three technical repetitions were performed for each sample). The Annexin V-fluorescein isothiocyanate (FITC)/propidium iodide (PI) apoptosis kit (Multi Sciences, Hangzhou, China) was used to detect cell apoptosis per the manufacturer’s instructions. After washing with PBS, the collected cells were resuspended with 1 × binding buffer. Next, 5 μL Annexin V-FITC and 10 μL PI were added, mixed, and incubated at room temperature for 5–10 min in the dark; 400 μL 1 × Binding Buffer was then added and mixed, and the samples were filtered with a 300-mesh cell sieve and tested on a BriCyte E6 flow cytometer (Mindray, Shengzhen, China) (n = 3, three technical repetitions were performed for each sample).

### 4.3. Oil Red O Staining and Immunofluorescence Assay

Oil red O staining was performed using a kit (Solarbio, Beijing, China) as per the manufacturers’ instructions. The steps for immunofluorescence of cells followed the method described by Chen et al. [16], using antibodies described in Supplementary Table S5.

### 4.4. Heat Stress Induction

Primary hepatocyte cells were incubated at 42 °C in a 5% CO_2_ incubator for 1 h, then allowed to recover at 37 °C. Cells were harvested immediately (0 h) or 6 h later, and then processed for qRT-PCR analysis (n = 3, three technical repetitions were performed for each sample). For other analyses, normally cultured primary preadipocytes were transferred to a 42 °C 5% CO_2_ incubator for 1, 2, or 3 days, and then collected (n = 3, three technical repetitions were performed for each sample).

### 4.5. m6A RNA Methylation Quantification

Relative mRNA m6A methylation was quantified using a EpiQuik m6A RNA methylation quantification kit (Epigentek, St. Louis, MO, USA). Briefly, the principal operational steps were as follows: (1) buffer and solution preparation; (2) RNA binding; (3) m6A RNA capture; and (4) m6A calculation. Calculation of the percentage of m6A in total RNA was carried out using the following formula: m6A %$=\frac{\left(Sample\;OD-NC\;OD\right)÷S}{\left(PC\;OD-NC\;OD\right)÷P}$, where S is the amount of input sample RNA in ng, P is the amount of input positive control (PC) in ng, and NC is native control (n = 3, three technical repetitions were performed for each sample).

### 4.6. Lentiviral Overexpression and RNAi Constructs and Infection of Cells

The target sequence for short hairpin RNA (shRNA) interference of *METTL3* was 5′-GAGAGCCTTCTTAACCAACAA-3′, and that for interference of *FTO* was 5′-ACGGTGAAATCTCTTTGAAAT-3′. Primer sequence information on the METTL3 and FTO overexpression constructs is shown in Supplementary Table S6. The synthesis and packaging of lentiviruses and the NC were performed by Genepharma Biotech Co (Shanghai, China).

For overexpression and interference experiments, primary hepatocytes were washed with PBS and then transfected with *METTL3* overexpression (M3-OE), *FTO* overexpression (FTO-OE), *METTL3* shRNA (M3-sR), *FTO* shRNA (FTO-sR), or NC lentivirus constructs for 72 h. Cells were isolated, and transfection efficiency was confirmed by qRT-PCR (n = 3, three technical repetitions were performed for each sample).

### 4.7. RNA-Seq Data Analysis

The cells were used to extract the total RNA using a TRIzol reagent kit (Invitrogen, Carlsbad, CA, USA) per the manufacturer’s protocol (n = 3). RNA quality was assessed on an Agilent 2100 Bioanalyzer (Agilent Technologies, Palo Alto, CA, USA) and checked using RNase-free agarose gel electrophoresis. After extracting the total RNA, eukaryotic mRNA was enriched with Oligo (dT) beads, and prokaryotic mRNA was enriched by removing the rRNA using a Ribo-Zero™ magnetic kit (Epicentre, Madison, WI, USA). The enriched mRNA was fragmented into short fragments using fragmentation buffer and reverse-transcribed into cDNA with random primers. Second-strand cDNA was synthesized using DNA polymerase I, RNase H, dNTP and buffer. The cDNA fragments were purified using a QiaQuick PCR extraction kit (Qiagen, Venlo, The Netherlands), end-repaired, A-base-added, and ligated to Illumina sequencing adapters. The ligation products were size selected via agarose gel electrophoresis, PCR amplified, and sequenced by OE Biotec Co., Ltd. (Shanghai, China) using an Illumina NovaSeq6000. The raw reads were filtered, and the clean reads were mapped to the reference sequences using HISAT2.2.4 software [50]. Gene expression levels were calculated using the FPKM method (fragment per kilobase of transcript per million mapped reads). Finally, differential expression analysis between two groups of RNA was performed using DESeq2 1.22.2 software [51]. Genes/transcripts with *p* < 0.05 and |log-fold change| ≥ 1 were considered DEGs. GO and KEGG pathway enrichment analyses of the DEGs were performed using R based on hypergeometric distribution. GO terms and KEGG pathways with *p* < 0.05 were considered significantly enriched.

### 4.8. RNA Extraction, Reverse Transcription, and qRT-PCR

A TransGen Biotech reverse transcription kit (Transgen, Beijing, China, refer to the instructions for specific methods) was used to reverse-transcribe extracted RNA into cDNA at 42 °C for 15 min and 85 °C for 5 s. The cDNA was stored at −20 °C. qRT-PCR was performed in 20 μL volumes per the manufacturer’s protocol (TransStar Tip Green Qpcr SuperMix, Transgen) on a Bio-Rad C1000 Thermal Cycler using the following conditions: 94 °C for 30 s and 40 cycles of 94 °C for 5 s, 60 °C for 15 s, and 72 °C for 10 s. *β-actin* was used as a reference gene to normalize gene expression. Supplementary Table S7 lists the primer sequences used for qRT-PCR.

### 4.9. Statistical Analysis

Statistical analyses were performed using one-way ANOVA by SPSS 22 software. The results were displayed as mean ± STD by GraphPad Prism 8 software. *p* < 0.05 was considered significant.

## 5. Conclusions

Heat stress promoted the expression of m6A methylation-modification genes and increased m6A RNA methylation levels in primary hepatocytes and preadipocytes derived from sheep. The expression levels of *HSP60*, *HSP70*, and *HSP110* were significantly downregulated, and the mRNA m6A methylation level was significantly increased under the overexpression of *METTL3* in primary hepatocytes; however, the interference of *FTO* reduced the methylation level of m6A. Primary adipocytes showed similar results. Transcriptome analysis of cells under *METTL3* (or *FTO*) inference and overexpression revealed differentially expressed genes enriched in the MAPK signaling pathways and PI3K-Akt and Ras signaling pathways. We speculate that *METTL3* may increase the level of m6A methylation to inhibit fat deposition and/or inhibit the expression of HSP genes to enhance the body’s resistance to heat stress, while the *FTO* gene generated the opposite molecular mechanism.

## Data Availability

The data presented in this study are deposited in the SRA repository, accession number: PRJNA988822, and our data have been released.

## Supplementary Materials

The following supporting information can be downloaded at: https://www.mdpi.com/article/10.3390/ijms241511926/s1.

## Abbreviations

AFP	Alpha-fetoprotein
ALKBK5	Alk B homologue 5
CCK-8	Cell Counting Kit-8
DEGs	Differentially expressed genes
FTO	Fat mass and obesity associated protein
GO	Gene Ontology
HS	Heat stress
HSP	Heat stress protein
KEGG	Kyoto Encyclopedia of Genes and Genomes
m6A	N6-methyladenosine
METTL3	Methyltransferase-like protein 3
METTL14	Methyltransferase-like protein 14
qRT-PCR	Quantitative reverse transcriptase-PCR
RNAi	RNA interference
WTAP	Wilms tumor 1 associated protein
YTHDF1	YTH domain family proteins 1
YTHDF2	YTH domain family proteins 2
YTHDC1	YTH domain containing proteins 1
YTHDC2	YTH domain containing proteins 2
