# Supplementary material for: Molecular Mechanism of m6A Methylation Modification Genes METTL3 and FTO in Regulating Heat Stress in Sheep
Source: Int J Mol Sci. 2023 Jul 25;24(15):11926. doi: 10.3390/ijms241511926 (PMC10419070; doi:10.3390/ijms241511926)
Supplement: Supplementary file 1 [file ijms-24-11926-s001.zip › ijms-2503857-supplementary.pdf]

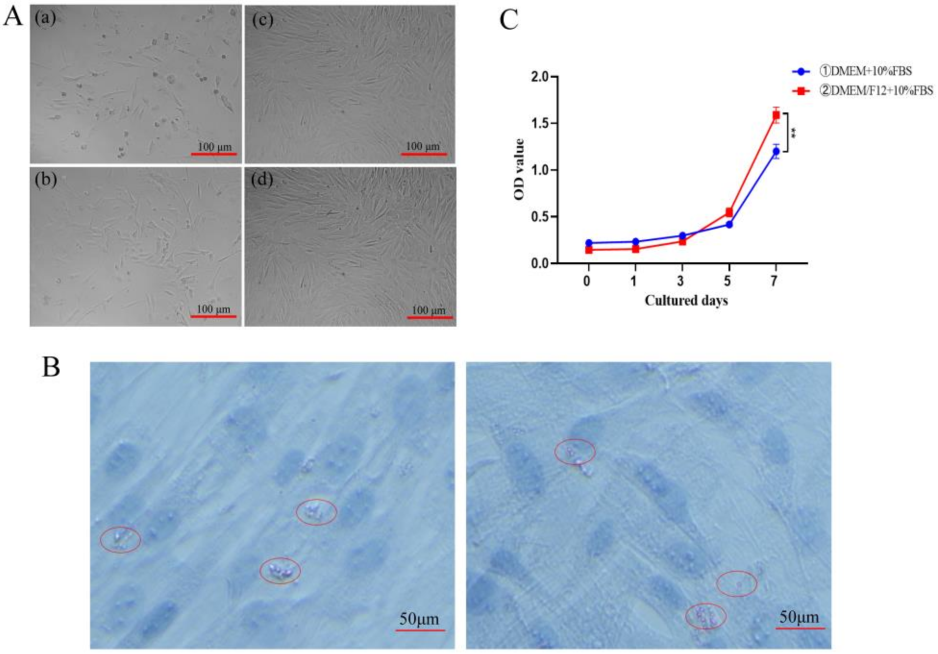

Figure S1 Morphological characteristics and proliferation detection of primary adipocytes. (A) Morphological characteristics of primary adipocytes. (a) Passage 0 (cultured 2 d); (b) Passage 0 (cultured 4 d)； (c) Passage 3; (d) Passage 4. (B) Oil red O staining; red circles represented tiny lipid droplets. (C) CCK-8 assay results of the proliferation of primary adipocytes. Notes: ** showed significant difference ($P < 0.05$).

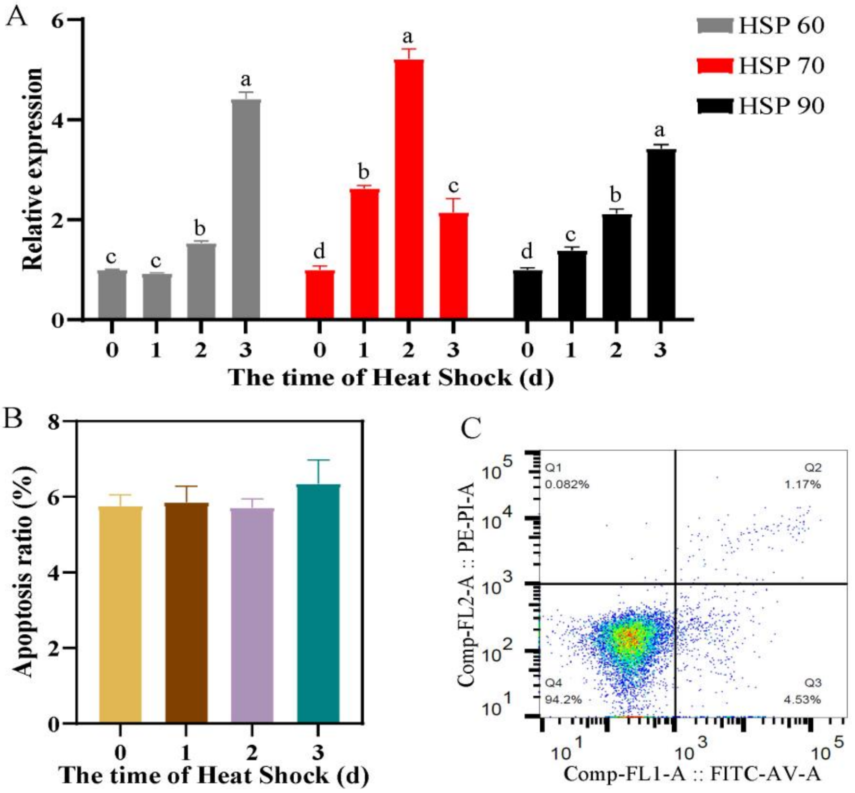

Figure S2 Detection of heat shock protein and apoptosis rate of primary adipocytes. (A) Expression of heat shock protein for *HSP60*, *HSP70*, and *HSP90*; (B) Statistical chart of apoptosis rate of primary adipocytes; (C) The apoptosis rate schematic diagram of primary adipocytes for no

HS. Groups marked with the same letter were considered to have no significant difference, and those without the same letter were significantly different ($P < 0.05$).

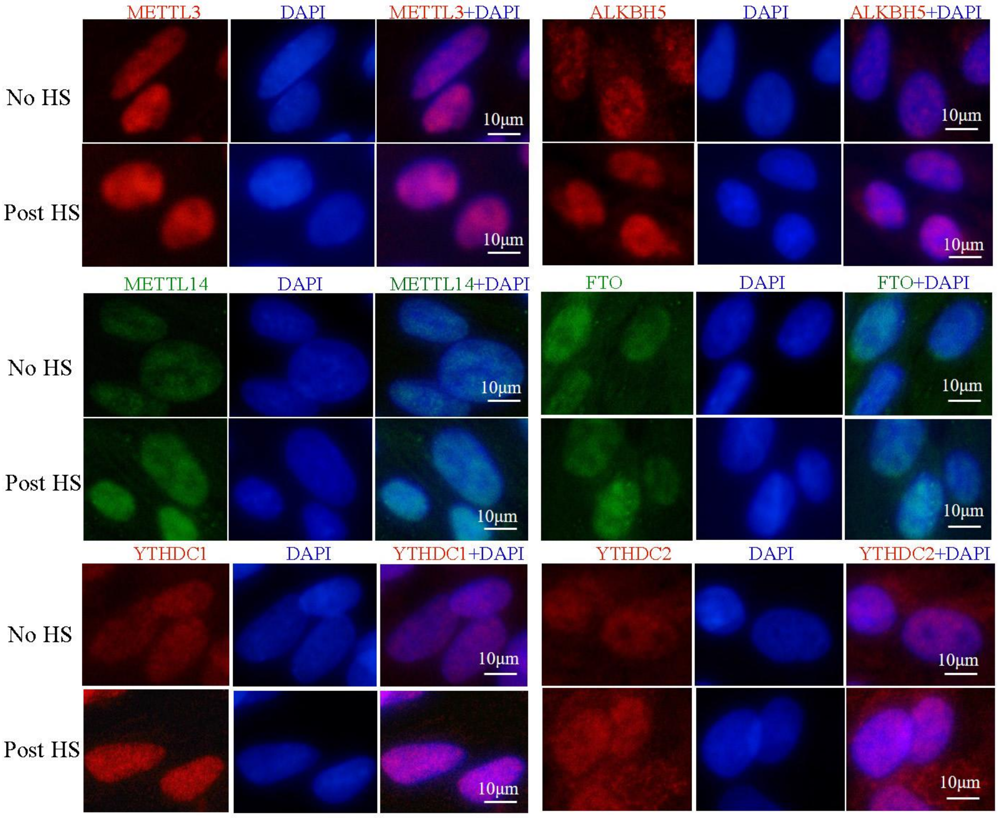

Figure S3 Immunofluorescence assays of no HS and post HS in primary hepatocytes. METTL3, ALKBH5, YTHDC1 and YTHDC2 are indicated with red fluorescence, METTL14 and FTO is green fluorescence and DAPI with blue.

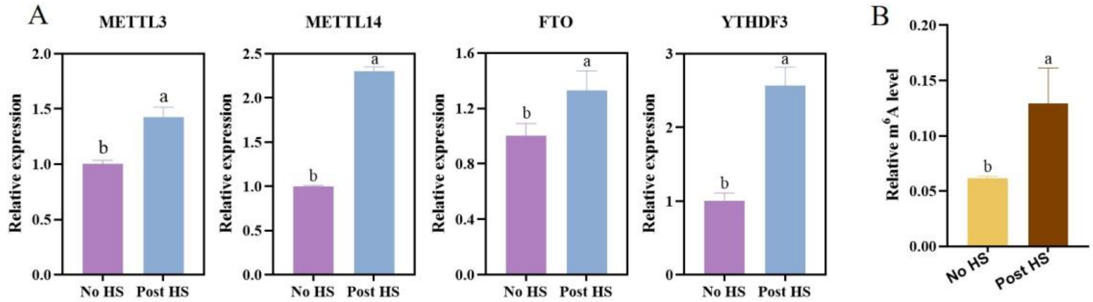

Figure S4 Detection of heat shock gene expression changes and m6A methylation levels of no HS and post HS in primary preadipocytes. (A) qRT-PCR analysis of *METTL3*, *METTL14*, *FTO* and *YTHDF3* gene expression; (B) mRNA m6A methylation quantification of no HS and post HS in primary preadipocytes. Groups marked with the same letter were considered to have no significant difference, and those without the same letter were significantly different ($P < 0.05$).

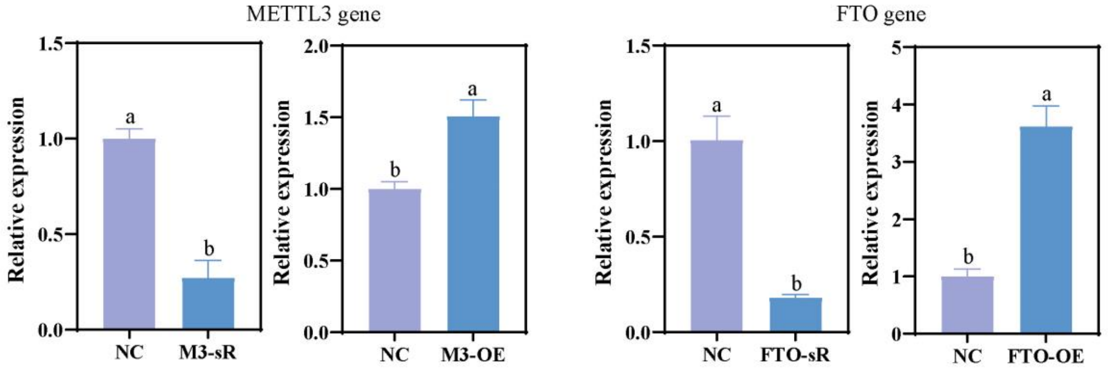

Figure S5 Interference and overexpression efficiency detection of *METTL3* and *FTO* gene. Groups marked with the same letter were considered to have no significant difference, and those without the same letter were significantly different ($P < 0.05$).

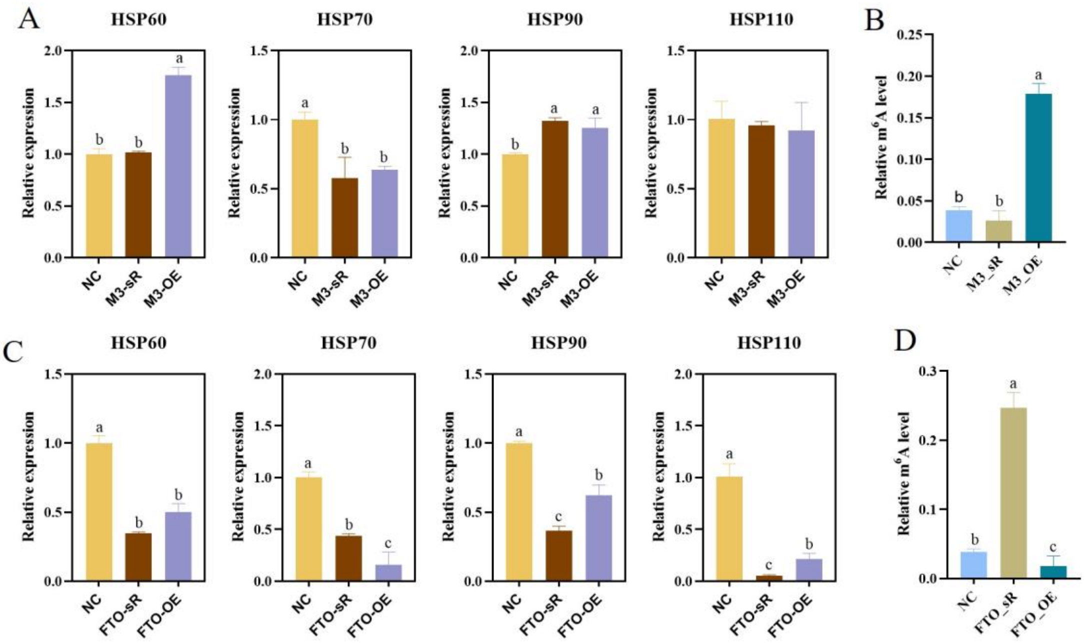

Figure S6 Detection of heat shock gene expression changes and m6A methylation levels of *METTL3* and *FTO* interference and overexpression in primary preadipocytes. (A) qRT-PCR analysis of heat shock gene expression change of *METTL3* interference and overexpression; (B) mRNA m6A methylation quantification of *METTL3* interference and overexpression; (C) qRT-PCR analysis of heat shock gene expression change of *FTO* interference and overexpression; (D) mRNA m6A methylation quantification of *FTO* interference and overexpression. Groups marked with the same letter were considered to have no significant difference, and those without the same letter were significantly different ($P < 0.05$).
