# Supplementary material for: Molecular Mechanism of m6A Methylation Modification Genes METTL3 and FTO in Regulating Heat Stress in Sheep
Source: Int J Mol Sci. 2023 Jul 25;24(15):11926. doi: 10.3390/ijms241511926 (PMC10419070; doi:10.3390/ijms241511926)
Supplement: Supplementary file 1 [file ijms-24-11926-s001.zip › Supplements Tables S1-S7/Table S1.pdf]

Table S1 Summary of the sequencing reads alignment to the reference genome

Sample	RawReads (M)	CleanReads (M)	Q30 (%)	GC (%)
Control1	49.20	47.66	96.62	54.43
Control2	49.13	47.50	96.24	54.81
Control3	48.35	47.00	96.28	54.80
HS1	50.92	48.81	96.85	51.59
HS2	50.86	48.52	96.59	51.54
HS3	51.30	48.76	96.60	51.57
M3_OE1	50.56	48.43	96.84	51.46
M3_OE2	51.58	48.90	96.90	51.39
M3_OE3	50.08	47.86	96.99	51.50
M3_sR1	50.14	48.30	96.29	51.63
M3_sR2	52.73	49.13	97.00	51.95
M3_sR3	49.95	48.09	96.76	51.96
FTO_OE1	51.49	49.12	96.92	51.69
FTO_OE2	51.59	48.83	96.80	51.17
FTO_OE3	51.92	49.18	96.95	51.20
FTO_sR1	50.27	48.08	96.73	51.92
FTO_sR2	50.96	48.50	97.00	51.94
FTO_sR3	51.08	48.55	97.03	51.72
