# Supplementary material for: Molecular Mechanism of m6A Methylation Modification Genes METTL3 and FTO in Regulating Heat Stress in Sheep
Source: Int J Mol Sci. 2023 Jul 25;24(15):11926. doi: 10.3390/ijms241511926 (PMC10419070; doi:10.3390/ijms241511926)
Supplement: Supplementary file 1 [file ijms-24-11926-s001.zip › Supplements Tables S1-S7/Table S5.pdf]

Table S5 Summary of the information of antibody

Antibody Name	Manufacturer	Host	Dilution Rate
METTL3	Proteintech	rabbit	1:200
METTL14	Proteintech	Mouse	1:200
FTO	Proteintech	mouse	1:100
WTAP	Proteintech	mouse	1:100
YTHDC1	Proteintech	rabbit	1:200
YTHDC2	Proteintech	rabbit	1:50
YTHDF1	Proteintech	rabbit	1:400
YTHDF1	Proteintech	rabbit	1:50
YTHDF3	Proteintech	rabbit	1:50
ALKBH5	Proteintech	rabbit	1:200
