# Supplementary material for: Molecular Mechanism of m6A Methylation Modification Genes METTL3 and FTO in Regulating Heat Stress in Sheep
Source: Int J Mol Sci. 2023 Jul 25;24(15):11926. doi: 10.3390/ijms241511926 (PMC10419070; doi:10.3390/ijms241511926)
Supplement: Supplementary file 1 [file ijms-24-11926-s001.zip › Supplements Tables S1-S7/Table S6.pdf]

Table S6 Primer sequence information of *METTL3* and *FTO* genes

METTL3:

B9711-1	AGGGTTCCAAGCTTAAGCGGCCGCGCCACCATGTCGGACACGTGGAGCTCTATCCAGGCCCA
B9711-2	TCCGCCTCCGCTGCAGCCTCTCCCGCAGTGAGTCCAGCTGCTTTTTGTGGGCCTGGATAGAGCTC
B9711-3	GCAGCGGAGGCGGAAGCAGGACTCGGGGCACTTGGATCTTCGGAATCCAGAGGCAGCACTGTCTC
B9711-4	ACCAGAAGTGGGTGCAGCAGGTACTGGGCTGTCGCTACGGAAGGTTGGAGACAGTGCTGCCTCTG
B9711-5	GCTGCACCCACTTCTGGTGGCCCCAAGCCCAGTACAGCTTCAGCAGTTCCTGAACTAGCTACAGA
B9711-6	CAGATCAGAGAGGTGGTGTAGCAACTTCTTCTCTAATTCAGGGTCTGTAGCTAGTTCAGGAACTG
B9711-7	CTACACCACCTCTCTGATCTGTCACTAACATTGCCCACTGATGCTGTGTCTATCCGTCTTGCCAT
B9711-8	AGGCTTTCTACCCCATCCTGAGTGGCAGGGGCATCTGGCGTGGAGATGGCAAGACGGATAGACAC
B9711-9	AGGATGGGGTAGAAAGCCTCCTACAGAAGTTTGCCGCTCAGGAGTTGATCGAAGTAAAGCGAAGT
B9711-10	CAGCATAGGTCACAAGAGTGGGATGTGCATCGTCTTGTAGGAGACTTCGCTTTACTTCGATCAAC
B9711-11	CCACTCTTGTGACCTATGCTGATCATTCCAAGCTCTCCGCCATGATGGGTGCTGTGGCAGAAAAG
B9711-12	GCCGCTTCTGCCCTGCAATAGTCCCTGCTACCTCCCCAGGGCCCTTCTTTTCTGCCACAGCACCC
B9711-13	CAGGGCAGAAGCGGCGTGCAGAGCAGGACTCCACCACAGCAGCTGCCTTTACTAGCTCTTTGGCC
B9711-14	CTTTGGCTCCTTGGCTACTTCTGATGTGGAAGAGGCCAGACCAGAGGCCAAAGAGCTAGTAAAGG
B9711-15	AAGTAGCCAAGGAGCCAAAGAAATCAAGAAAACATGCTGCCTCAGATGTTGATCTGGAGATAGAG
B9711-16	TTGCTCTGTTGTTCCTTAGTAGATTGTTGGTTAAGAAGGCTCTCTATCTCCAGATCAACATCTGA
B9711-17	TCTACTAAGGAACAACAGAGCAAGAAGGTTAGTCAGGAGATCCTAGAGTTACTAAATACTACAAC
B9711-18	GAGCGAAACTTTTCAACAATGGATTGTTCCTTGGCTGTTGTAGTATTTAGTAACTCTAGGATCTC
B9711-19	CCATTGTTGAAAAGTTTCGCTCACGAGGTCGGGCTCAAGTTCAAGAGTTCTGTGACTATGGAACC
B9711-20	GACAGGGCCGGTCAGCATCACTGGCTTTCATACACTCCTCCTTGGTTCCATAGTCACAGAACTCT
B9711-21	GCTGACCGGCCCTGTCGAAAGCTGCACTTCAGACGGATCATCAATAAACACACTGATGAGTCATT
B9711-22	ATCCATGTGGAAACATGTGTTAAGGAAAGAGCAGTCTCCTAATGACTCATCAGTGTGTTTATTGA
B9711-23	TTAACACATGTTTCCACATGGATACCTGCAAATATGTTCACTATGAAATTGATGCTTGCATGGAT
B9711-24	GCTCTTGGCTTGGTGTATGGTCTTTGCTTCCAGGAGCCTCAGAATCCATGCAAGCATCAATTTCA
B9711-25	CCATACACCAAGCCAAGAGCTTGCCCTTACACAGAGCGTTGGAGGTGACTCCAATGCAGATCGAC
B9711-26	CGTCCAGGTAGCGGATATCACAACAGATCCACTGAGGTGGGAAGAGTCGATCTGCATTGGAGTCA
B9711-27	TGATATCCGCTACCTGGACGTCAGTATCTTGGGCAAGTTTGCAGTTGTGATGGCTGACCCACCCT

B9711-28	TCTCATCATCTGTCAGGGTCCCATAGGGCAGCTCCATGTGAATATCCCAGGGTGGGTCAGCCATC
B9711-29	GACCCTGACAGATGATGAGATGCGCAGGCTCAACATACCAGTACTGCAGGATGATGGCTTTCTCT
B9711-30	GACATTCTCTGCCCAACTCCATGGCCCTGCCTGTGACCCAGAGGAAGAGAAAGCCATCATCCTGC
B9711-31	GGAGTTGGGCAGAGAATGTCTGAACCTCTGGGGTTATGAACGGGTAGATGAAATTATCTGGGTGA
B9711-32	TGTGCGGCCTGTTCGAATGATGCGCTGCAGTTGATTTGTCTTCACCCAGATAATTTCATCTACCC
B9711-33	TTCGAACAGGCCGCACAGGTCACTGGTTGAACCATGGGAAGGAACACTGCTTGGTTGGTGTCAAA
B9711-34	GATCACATCACAATCCAGACCCTGGTTGAAGCCTTGGGGATTTCCTTTGACACCAACCAAGCAGT
B9711-35	GGGTCTGGATTGTGATGTGATCGTAGCTGAGGTTCGTTCCACTAGTCATAAACCAGATGAAATCT
B9711-36	ATCTTGCGAGTGCCAGGGGACAGTCTTTCAATCATGCCATAGATTTCATCTGGTTTATGACTAGT
B9711-37	CCCTGGCACTCGCAAGATTGAGTTATTTGGACGACCACACAATGTGCAACCCAACTGGATCACCC
B9711-38	ACCACATCTGGGTCTAGTAGGTGGATCCCATCCAATTGGTTTCCAAGGGTGATCCAGTTGGGTTG
B9711-39	CCTACTAGACCCAGATGTGGTTGCCCGGTTCAAGCAAAGATATCCAGATGGTATCATCTCTAAAC
B9711-40	GATCCATCCCTAGGTAGATGCATCTATAGATTCTTAGGTTTAGAGATGATACCATCTGGATAT

FTO:

B9713-1	AGGGTTCCAAGCTTAAGCGGCCGCGCCACCATGAAGCGGACCCCGACGGCCGAGGAACGAGAGC
B9713-2	AGCCATGTGTCTTCAAGCTCTTCAAGAAGCCTCAGTTTCTTAGCTTCGCGCTCTCGTTCCTCGGC
B9713-3	GAGCTTGAAGACACATGGCTTCCCTACCTGACCCCCAAAGACGATGAATTCTATCAGCAGTGGCA
B9713-4	GCACGCTGGCAGCTTCTCGGAGAATCAGTTTAGGATATTTCAGTTGCCACTGCTGATAGAATTCA
B9713-5	GAAGCTGCCAGCGTGCCCGAGTTGCTCCATAAAGAGGTTCAACAAGCCTTTCTCACACTGCACAA
B9713-6	TCTTTGCCCTGGATCCTCACCAGATCTCGAAATAAACAGCCATGCTTGTGCAGTGTGAGAAAGGC
B9713-7	TGAGGATCCAGGGCAAAGACTTGCTCACTCCAGTCTCTCGCATCCTCATCGGTAACCCTGGCTGC
B9713-8	CACGGGCCAGGGTACCGTGAAGAGCCTGGTGTTCAGGTACTTGTAGGTGCAGCCAGGGTTACCGA
B9713-9	GGTACCCTGGCCCGTGAAAGGCTCTGATGCAAAGTACAATGAGGCCGAAATAGCTGTCGCCTGTC
B9713-10	GGATGGTCTCTACCTGCAGGTAACTGTTGAGCTTGAGGAAGGTTTGACAGGCGACAGCTATTTCG
B9713-11	CTGCAGGTAGAGACCATCCAGGCTTTGGAAGAGCTTGCTGCCAAGGAGAAAGCCAACATCGATGC
B9713-12	CCCATGCCAACCCTGGGGAAATCTGGACCTATGCACACTGGCACAGCATCGATGTTGGCTTTCTC
B9713-13	CCAGGGTTGGCATGGGGTCCTCCTTTGACGGGCACGATGAGATTGACATGAAGAACCGAGCAGCG
B9713-14	TACGGCATTTTCTGGGGATCCATGAAATTCAACAAAGTGACGTTGTACGCTGCTCGGTTCTTCAT

B9713-15	GATCCCCAGAAAATGCCGTACCTGAAAGAGGAACCATATTTCGGCATGGGGAAAATGGCCGTGAG
B9713-16	ACACTGCCACCGCTGACCTGTCCACCAGATTTTCATCATGATGCCAGCTCACGGCCATTTTCCCC
B9713-17	TCAGCGGTGGCAGTGTACAGTTATAGCTGTGAAGGCCCTGAAGAGGAAAGTGAGGATGACCCTCA
B9713-18	ATCTTAAAACCAACGTGCCAAATATCAGGATCTCTGCCTTCAAGCTGAGGGTCATCCTCACTTTC
B9713-19	TTGGCACGTTGGTTTTAAGATCTCGTGGGACATAGAGACACCTGGGTTGGCGATACCCCTTCACC
B9713-20	TTGGTGGGTGGCATTGAGATCATCAAGCATAAAATAGCAGTCTCCTTGGTGAAGGGGTATCGCCA
B9713-21	CTCAATGCCACCCACCAACACTGTGTTTTGGCTGGTTTACCACCTCGCTTTAGTTCCACCCACCG
B9713-22	GCGCTGTAAGATGTATTCCAAGGTCCCCGTCGAGCACTCTGCCACTCGGTGGGTGGAACTAAAGC
B9713-23	CTTGGAATACATCTTACAGCGCTGCCAGGTGGCCCTGCAGAATGTCCGCGAAGAGGCAGACAACG
B9713-24	TCCTTGTTTCAAAACCACTGACTCCAAGGATTTCAAAGAGATTTCACCGTTGTCTGCCTCTTCGC
B9713-25	GTCAGTGGTTTTGAAACAAGGAGAAGAGATCCACAACGAGGTCGAGTTTGAATGGCTGAGACAGT
B9713-26	CCACCAGTCAGTGCACTTTTTGTATCGACTGCCTTGAAACCAGAACTGTCTCAGCCATTCAAACT
B9713-27	AAAGTGCACTGACTGGTGGTGTCAGCCCATGAGTCAGCTGGAAGAGATGTGGAGAAAGATGGAGT
B9713-28	ACCCCCTCTCTTCTAACTTCACGAAGCACAGCGCTTGTCAGCCACTCCATCTTTCTCCACATCTC
B9713-29	TGAAGTTAGAAGAGAGGGGGTCCCCATGGAACAAAAGAATGAGATGTTGACGTCCATCCTCGCCT
B9713-30	GGCACCTGGCATGCCATTCTCTCCTCAGGTTCTGGCGGGTGGTGATCGAGGCGAGGATGGACGTC
B9713-31	TGGCATGCCAGGTGCCAGTCTCGAATTGCCCGGACTTTACCCGCTGATCAGAAGCCAGAGTGCCG
B9713-32	GGTCGAATGGCAGAGGCATCGAAGGATCACCCTTTTCCCAGTATGGCCGGCACTCTGGCTTCTGA
B9713-33	GCCTCTGCCATTCGACCTCACAGAGATCGTTTCTGAACTCAGAGGTCTGCTTCTGGAGACCAGGC
B9713-34	GATCCATCCCTAGGTAGATGCATCTAGGGCCTGGTCTCCAGAAGCA
