# Supplementary material for: Molecular Mechanism of m6A Methylation Modification Genes METTL3 and FTO in Regulating Heat Stress in Sheep
Source: Int J Mol Sci. 2023 Jul 25;24(15):11926. doi: 10.3390/ijms241511926 (PMC10419070; doi:10.3390/ijms241511926)
Supplement: Supplementary file 1 [file ijms-24-11926-s001.zip › Supplements Tables S1-S7/Table S7.pdf]

Table S7 Primer sequence information of qRT-PCR

| Gene | Primer sequences (5'-3') | Accession No. | Products length (bp) |
|---|---|---|---|
| *METTL3* | F: AGATAGAGAGCCTTCTTAACCA<br>R: TCACTGGCTTTCATACACTCC | XM_004010362.3 | 196 |
| *METTL14* | F: ATATTACAGAGAGACCGGCAT<br>R: CAATCCTTCCCCAGAACCACA<br>R: CTTCAGATTCCCTTAAGCCAGT | XM_004009592.3 | 133 |
| *FTO* | F: CATGGCTTCCCTACCTGACC<br>R: CTCGAAATAAACAGCCATGCTT | EU072419.1 | 165 |
| *YTHDF3* | F: CCTGTAATTCTACTCGTGGTGA<br>R: AAATATTCTGCTGCACGTTG | XM_015097785.1 | 200 |
| *HSP60* | F: CCTTAATGCTACACGAGCTG<br>R: AACACCTGCATTCTTAGCAA | XM_004004792.3 | 184 |
| *HSP70* | F: CCCACGAAGCAGACGCAGAT<br>R: GCAGGTTGTTGTCCCGAGTCAT | JN604434.1 | 106 |
| *HSP90* | F: CAGTTCATTGGCTATCCCAT<br>R: TCTTCATCTGAGCCAACGTCT | EF091713.1 | 164 |
| *HSP110* | F: ACAAGCTGGAAGAACTAACGAA<br>R: AGTCAGCTGCTATCTTGGCAT | XM_015098145.1 | 129 |
| *ACTB* | F: CTTCCAGCCTTCCTTCCTGG<br>R: GCCAGGGCAGTGATCTCTTT | NM_001009784.1 | 180 |
